# KIR2DL2/2DL3-E^35^ alleles are functionally stronger than -Q^35^ alleles

**DOI:** 10.1038/srep23689

**Published:** 2016-03-31

**Authors:** Rafijul Bari, Rajoo Thapa, Ju Bao, Ying Li, Jie Zheng, Wing Leung

**Affiliations:** 1 Department of Bone Marrow Transplantation and Cellular Therapy, St. Jude Children’s Research Hospital, Memphis, Tennessee, USA; 2 Department of Structural Biology, St. Jude Children’s Research Hospital, Memphis, Tennessee, USA; 3 Department of Pediatrics, University of Tennessee Health Science Center, Memphis, Tennessee, USA

## Abstract

*KIR2DL2* and *KIR2DL3* segregate as alleles of a single locus in the centromeric motif of the killer cell immunoglobulin-like receptor (KIR) gene family. Although KIR2DL2/L3 polymorphism is known to be associated with many human diseases and is an important factor for donor selection in allogeneic hematopoietic stem cell transplantation, the molecular determinant of functional diversity among various alleles is unclear. In this study we found that KIR2DL2/L3 with glutamic acid at position 35 (E^35^) are functionally stronger than those with glutamine at the same position (Q^35^). Cytotoxicity assay showed that NK cells from HLA-C1 positive donors with KIR2DL2/L3-E^35^ could kill more target cells lacking their ligands than NK cells with the weaker -Q^35^ alleles, indicating better licensing of KIR2DL2/L3^+^ NK cells with the stronger alleles. Molecular modeling analysis reveals that the glutamic acid, which is negatively charged, interacts with positively charged histidine located at position 55, thereby stabilizing KIR2DL2/L3 dimer and reducing entropy loss when KIR2DL2/3 binds to HLA-C ligand. The results of this study will be important for future studies of KIR2DL2/L3-associated diseases as well as for donor selection in allogeneic stem cell transplantation.

Killer cell immunoglobulin-like receptors (KIRs) are expressed on the surface of most NK cells and a subset of T-cells^1^,^2^. The expression of KIR genes varies stochastically so that a single NK cell may express one to a few KIRs^2^,^3^. There are 15 KIR genes but only approximately 5% of individuals have all 15 KIR genes; others lack one or more genes^4^,^5^. Haplotypic variability in gene content is the result of gene duplication and deletion throughout evolution^4^,^6^. For example, KIR2DL2 appears to be an evolutionally newer fusion gene formed by unequal crossing between the extracellular domains of KIR2DL3 and the intracellular tail of KIR2DL1^7^. Segregation analysis show that KIR2DL2 and KIR2DL3 actually behave as alleles at a single locus (KIR2DL2/3)^8^. Since their ligand-binding domains are highly homologous, KIR2DL2 and KIR2DL3 bind a similar set of HLA-C ligands. Functionally, it has been suggested that KIR2DL2 is a stronger receptor for HLA-C compared to KIR2DL3 in general^9^,^10^.

Twelve alleles have been identified for KIR2DL2 and 24 alleles for KIR2DL3 thus far. The allelic variations may generate further functional differences. For example, we have shown previously that KIR2DL1 alleles with arginine in position 245 of the transmembrane domain (KIR2DL1-R^245^) are stronger than those with cysteine (KIR2DL1-C^245^)^11^. In the Japanese population, 5 common KIR3DL1 allotypes, *001, *01502, *002, *005, and *007, have distinguishable inhibitory capacity, frequency of cellular expression, and level of cell-surface expression^12^. Carr *et al.* showed that the presence of arginine (R) at position 238 in the D2 domain and isoleucine at position 320 in the transmembrane domain of the KIR3DL1*002 allele makes it a stronger receptor than the *007 allele that has glycine (G) and valine (V) at those positions^13^.

Although the molecular determinants of KIR2DL2/L3 functional diversity have not been elucidated, Frazier *et al.* have shown differential affinity and avidity of some common KIR2DL2 and KIR2DL3 alleles toward their HLA-C ligands, using Surface Plasmon Resonance (SPR)^14^. Three KIR2DL2 alleles (*001, *003 and *006) showed higher ligand affinity and avidity when compared to two other KIR2DL3 alleles (*001 and *002) that had glutamine at amino acid position 35 (Q^35^). Surprisingly, one KIR2DL3 family member (*005) that contains glutamic acid at the same position (E^35^) showed high affinity and avidity toward HLA-C ligand at a level similar to those of KIR2DL2 alleles^14^. In this study, we hypothesized that these differences in affinity and avidity would translate to biologic relevance in NK cell function. We investigated whether position 35 could be a useful biomarker to distinguish stronger versus weaker KIR2DL2/L3 for clinical uses.

## Results

### KIR2DL2/L3-E^35^ is functionally stronger than -Q^35^

**Figure 1:**
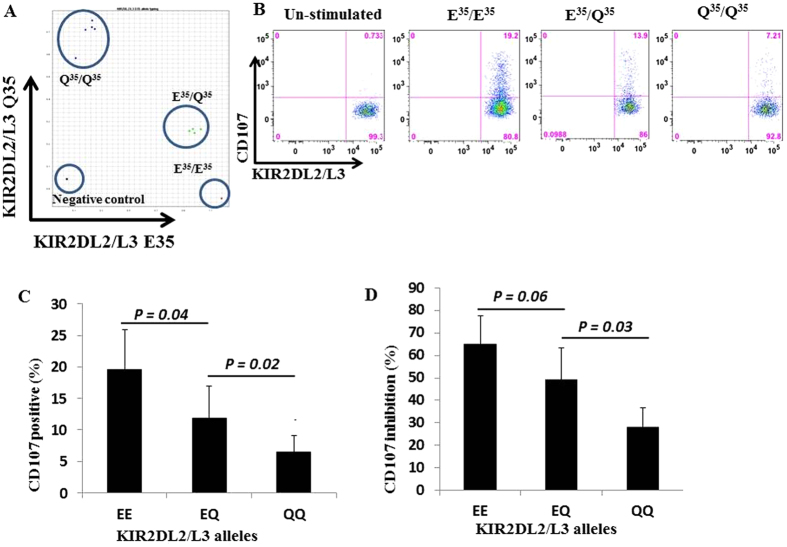
KIR2DL2/L3-E^35^ is functionally stronger than KIR2DL2/L3-Q^35^. (**A**) A single nucleotide polymorphism assay was developed to detect the presence of glutamic acid or glutamine at position 35 of KIR2DL2/L3 alleles. (**B**) Representative CD107 degranulation assays in which single KIR2DL2/L3^+^ NK cells were tested for missing-self recognition using 721.221 cells without C1 ligand. (**C**) Summary results of CD107 degranulation assay from 4–5 donors with each allelic group. (**D**) CD107 inhibition by different KIR2DL2/L3 allelic groups when stimulated with 721.221 cells expressing Cw07 ligand. Percentage indicates relative value when compared with target cells without Cw07 ligand. Results are average from 4–5 donors in each KIR2DL2/L3 allelic group. P values were calculated using Student T test. Error bars represent SD.

The majority of KIR2DL2 alleles have glutamic acid at position 35 (E^35^) except KIR2DL2*004, whereas approximately half of the KIR2DL3 alleles contain glutamine at the same position (Q^35^) (Table 1). KIR2DL2 was reported to have higher affinity towards HLA-C ligands and stronger function than KIR2DL3 in general^9^,^14^, however, KIR2DL3*005 allele with E^35^ has affinity and avidity towards their HLA-C ligands similar to those of KIR2DL2 alleles^14^. We therefore hypothesized that position 35 could be a molecular determinant of KIR2DL2/L3 functional strength. We developed a single nucleotide polymorphism (SNP) assay that has the ability to distinguish KIR2DL2/L3-E^35^ from KIR2DL2/L3-Q^35^. We typed 10 donors for the presence of alleles encoding KIR2DL2/L3-E^35^ and -Q^35^ (Fig. 1A) and validated the results by sequencing.

Next, we assessed the functional activities of single KIR2DL2/L3^+^ NK cell subset (i.e. KIR2DL1^−^KIR3DL1^−^NKG2A^−^) from 14 normal donors with different KIR2DL2/L3 allelic groups by CD107 degranulation assay. When stimulated with 721.221 cells lacking HLA-C ligands, KIR2DL2/L3-E^35^/E^35^ NK cells showed significantly higher degranulation than KIR2DL2/L3-Q^35^/Q^35^ NK cells (Fig. 1B,C). NK cells with KIR2DL2/L3-E^35^/Q^35^ showed intermediate degranulation (Fig. 1B,C). KIR2DL2/L3-E^35^/E^35^ NK cells also produced more interferon-γ (IFN-γ) and granzyme B than KIR2DL2/L3-Q^35^/Q^35^ cells when stimulated with 721.221 cells (Supplementary Figure S1).

When NK cells with different KIR2DL2/L3 allelic groups were mixed with 721.221 cell expressing HLA-Cw07 ligands, KIR2DL2/L3-E^35^/E^35^ NK cells showed significantly greater inhibition of CD107 degranulation than KIR2DL2/L3-Q^35/^Q^35^ NK cells (Fig. 1D). KIR2DL2/L3-E^35^/Q^35^ NK cells had intermediate degranulation (Fig. 1D). These results suggest that KIR2DL2/L3-E^35^ is functionally stronger than KIR2DL2/L3-Q^35^ and there is a dosage effect.

### Functional differences between different KIR2DL2/L3 allelic groups are independent of self C1 ligand, KIR2DL2/L3 genotype or other NK receptor phenotype

**Figure 2:**
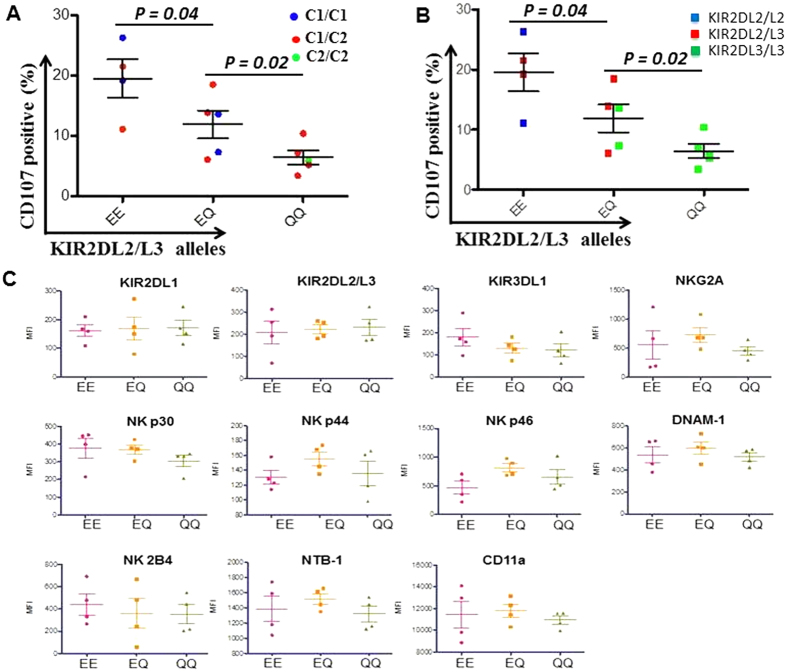
Functional differences between different KIR2DL2/L3 allelic groups are independent of self C ligand, KIR2DL2/L3 genotype, or other NK cell phenotype. (**A**) HLA-C ligands of 14 donors used for functional assay were genotyped using SNP assay and color coded. Blue 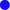 indicates HLA-C1 homozygous; red 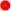 indicates HLA-C1/C2 heterozygous; green 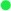 indicates HLA-C2 homozygous. (**B**) Donor KIR2DL2/L3 genotyping were performed using SSP-PCR and are shown similarly in color code. (**C**) NK cell phenotype of donors with different groups of KIR2DL2/L3 alleles were analyzed by flow cytometry. P values were calculated using Student T test. Error bars represent SD.

Since NK cell responsiveness to missing self is regulated by licensing effect of self-ligand^15^,^16^, we examined the HLA-C1/C2 genotypes among the 14 donors (Supplementary Figure S2). Of the 14 donors, one was C2/C2 and his KIR2DL2/L3^+^ NK cells had low CD107 degranulation (Fig. 2A), in part because the cells were unlicensed and had the weakest Q^35^/Q^35^ combination. Of the other 13 C1^+^ donors, the level of CD107 degranulation appeared to follow the pattern of EE > EQ > QQ, rather than C1C1 > C1C2. These results suggest that the observed functional differences in E^35^ and Q^35^ may be independent of ligand effect, although a larger number of individuals in each group will need to be studied to rule out an effect of the HLA-C haplotypes.

Because the most common KIR2DL2 alleles contain E^35^ and KIR2DL3 alleles contain Q^35^ (Table 1), we sought to determine whether it is clinically sufficient to genotype donors as KIR2DL2/L2, KIR2DL2/L3 and KIR2DL3/L3 (corresponding to centromeric B score of 2, 1, 0, respectively), rather than grouping them into EE, EQ and QQ respectively. To address this question, we genotyped KIR2DL2 and KIR2DL3 using SSP-PCR^17^. Of the 4 EE donors, 2 were KIR2DL2/KIR2DL2 and 2 were KIR2DL2/KIR2DL3 (Fig. 2B,). All the QQ donors were KIR2DL3/KIR2DL3. Of the 5 EQ donors, 3 were KIR2DL2/KIR2DL3 and 2 were KIR2DL3/KIR2DL3. These results suggest that E^35^ is common in many KIR2DL3 alleles and E^35^/Q^35^ SNP analysis is essential for KIR2DL2/L3 functional typing.

Besides KIR2DL2/L3, NK cells may express many other activating and inhibitory receptors that can also influence their functions. In this regard, Schonberg *et al.* reported that the presence of KIR2DL2 causes a major reduction in the frequency of NK cells expressing KIR2DL1 independent of the presence or absence of the C1 or C2 epitope^10^. Therefore, we analyzed the expression of various NK cell receptors in donors with different allelic groups of KIR2DL2/L3. Although there was natural variation in the expression of different NK receptors among different donors, we did not observe any statistically significant association with different allelic groups of KIR2DL2/L3 (Fig. 2C). Thus, the functional difference between E^35^ and Q^35^ alleles could not be accounted by linkage disequilibrium with other NK cell receptor expression.

### Substitution of glutamic acid with glutamine significantly reduces the functional strength of KIR2DL2*001

**Figure 3:**
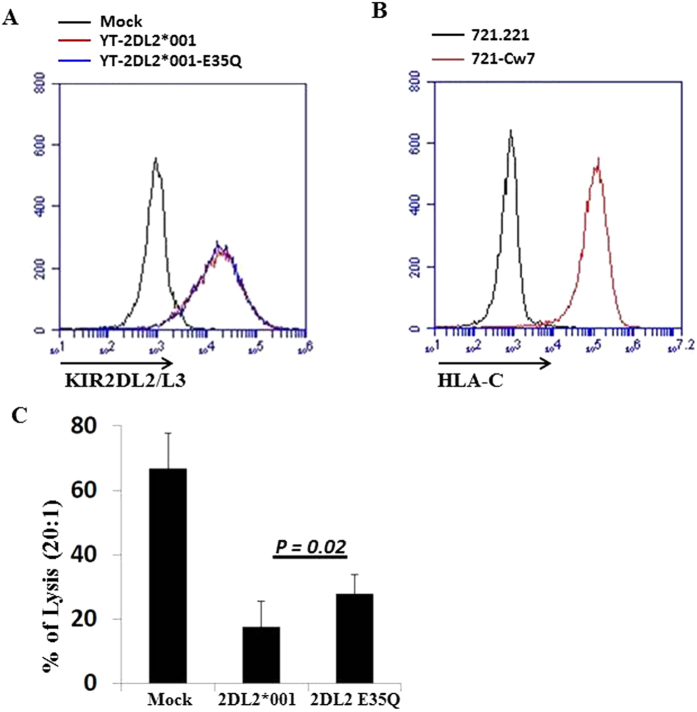
Substitution of glutamic acid with glutamine at position 35 significantly reduces the functional strength of KIR2DL2*001. (**A**) Stable YT-Indy cell lines expressing KIR2DL2*001 or KIR2DL2*001 mutant with glutamic acid at the amino acid position 35 replaced with glutamine. YT-Indy transfected with empty vector was used as mock control. Similar expression of KIR2DL2/L3 in the cell lines was confirmed by flow cytometry. (**B**) 721.221 was stably transduced with MSCV vector containing HLA-Cw07 ligand. (**C**) Specific killing of YT-Indy expressing KIR2DL2*001 or KIR2DL2*001 mutants (2DL2 E^35^Q) were assessed against 721.221-Cw07 by BADTA release assay. The experiments were repeated at least 3 times. P value was calculated using Student T test. Error bars represent SD.

To directly demonstrate the effect of E^35^/Q^35^ polymorphism without potential influence from other known and unknown receptors expressed on polyclonal NK cells, monoclonal YT-Indy cell line was transfected with KIR2DL2*001 or a KIR2DL2*001 mutant with glutamine substituting glutamic acid at position 35 (KIR2DL2*001 E35Q). Stable cell lines with similar level of KIR2DL2/L3 expression were sorted by flow cytometer (Fig. 3A) and subjected to further functional analysis. Stable 721.221 cell line transduced with HLA-Cw07 (721-Cw7) ligands (Fig. 3B) was used as target cells. KIR2DL2*001 (E^35^) strongly inhibit the cytotoxicity of YT-Indy in the presence of HLA-Cw07 but the substitution of glutamic acid with glutamine at amino acid position 35 (E35Q) resulted in a weaker inhibitory receptor (Fig. 3C). As expected, there were no significant functional differences among mock, KIR2DL2*001 or a KIR2DL2*001 mutant against MHC-I deficient 721.221 cells (Supplementary Figure S3).

### *In vivo* anti-tumor activity of YT-Indy cells expressing different KIR2DL2/L3 alleles and mutants

**Figure 4:**
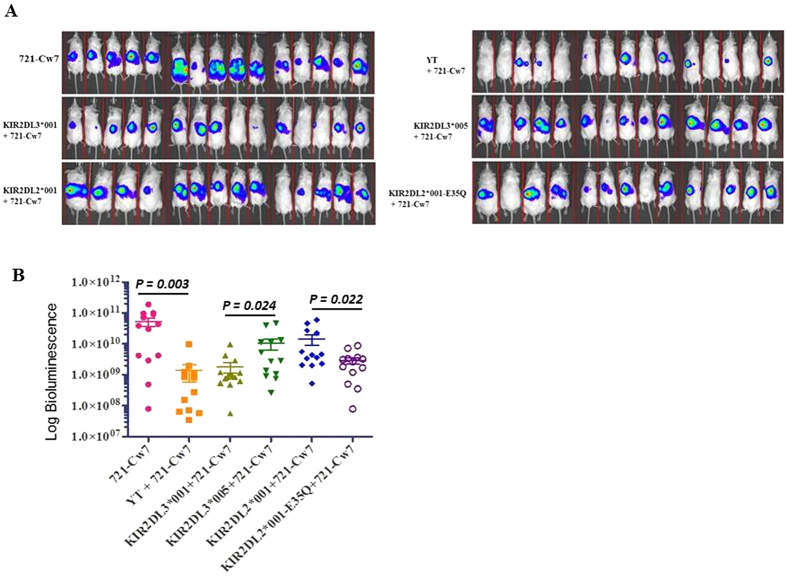
Effector cells expressing KIR2DL2/L3-E^35^ showed stronger inhibition than those with KIR2DL2/L3-Q^35^ allele *in vivo*. YT-Indy cell line expressing different KIR2DL2/L3-E^35^, -Q^35^ or mutant alleles were injected in NSG mice with 721.221 target cells expressing HLA-Cw7 ligand and luciferase. (**A**) Bioluminescence pictures of mice after two weeks of injection. (**B**) Comparison of average bioluminescence signals among different groups of mice injected with YT-Indy cells carrying different KIR2DL2/L3 alleles. P values were calculated using Student T test. Error bars represent SD.

A mouse model was used to assess the anti-tumor activity of YT-Indy cells expressing different KIR2DL2/L3 alleles against 721.221 target cells expressing HLA-Cw07 ligand and luciferase. On day one, 8- to 12-week-old NSG mice were injected intraperitoneally with 5 × 10^5^ target cells. Next day, YT-Indy cells expressing either KIR2DL3*001 (Q^35^), KIR2DL3*005 (E^35^), KIR2DL2*001 (E^35^) or KIR2DL2*001-E35Q mutant were injected at a 10:1 E/T ratio. After 2 weeks, the bioluminescence signals of the mice that were injected with target cells only showed the highest signal whereas the group injected with YT-Indy cells containing empty vector (mock) showed the lowest signal (Fig. 4A). KIR2DL3*001 (Q^35^) showed less inhibition than KIR2DL3*005 (E^35^). While KIR2DL2*001 (E^35^) showed strong inhibition (Fig. 4A,B), mutation of E^35^ to Q^35^ in KIR2DL2*001 markedly reduced inhibition to level similar to those of KIR2DL3*001 (Q^35^), suggesting that E^35^ is essential for durable inhibition *in vivo*.

### Molecular modeling analyses suggest glutamic acid at position 35 of KIR2DL2/L3 forms hydrogen bond with histidine at position 55

**Figure 5:**
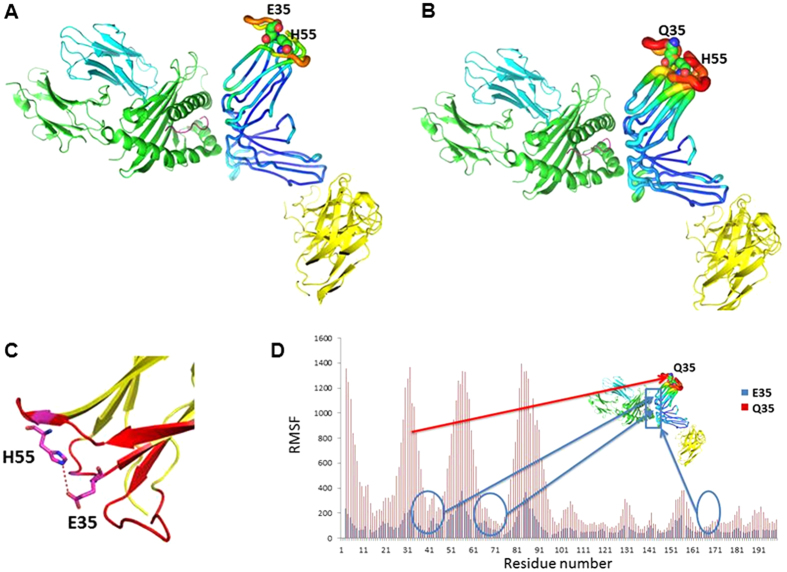
Structural effects of KIR2DL2-E^35^Q substitution. The effects of E^35^Q substitution in KIR2DL2 in MD simulations are shown in (**A**) KIR2DL2*001-E^35^ and (**B**) KIR2DL2*001-Q^35^. KIR monomer binding to HLA ligand is shown in sausage representation. The other KIR monomer and HLA are shown in cartoon representation. Larger radii and warmer color indicate higher flexibility. Residues at positions 35 and 55 are shown in sphere. E^35^Q not only significantly destabilizes its adjacent regions but also substantially increases the flexibility of HLA binding interface. (**C**) E^35^ is predicted to form hydrogen bond with H^55^ side chain, which may contribute to the stabilization of the KIR2DL2-E^35^ structure. (**D**) E^35^Q causes significant conformational flexibility change of KIR2DL2, shown by much higher RMSF of the mutant. E^35^Q substitution in KIR2DL2 greatly destabilizes the adjacent regions of the protein, and changes the flexibility of KIR2DL2 and HLA ligand binding site. E^35^Q and other residues that are involved in KIR2DL2 and HLA ligand interaction are mapped to the KIR2DL2-HLA structure. The HLA binding site on KIR2DL2 is highlighted with a blue frame.

To understand the structural mechanism, we performed molecular dynamic simulations on KIR2DL2*001-E^35^ and KIR2DL2*001-Q^35^ dimers. Overall, KIR2DL2*001-Q^35^ showed substantially higher flexibility compared to KIR2DL2*001-E^35^ (Fig. 5A,B). Amino acid position 35 of KIR2DL2/L3 is located in the loop region and does not directly interact with either HLA binding or dimerization interface. However, KIR2DL2/L3 alleles with E^35^ form strong electrostatic interactions with the side chain of histidine at position 55 (Fig. 5C), which significantly stabilized not only the loop regions but also the HLA binding interface. Destabilization of this region greatly increases the flexibility of both loop regions and HLA binding interface (Fig. 5D), which reduces the binding affinity of KIR2DL2/L3 with HLA by increasing entropy loss during binding. Since it has been shown earlier that KIR2DL2/L3 dimer stability significantly influences its binding affinity with its ligands, the simulation data supports the hypothesis that the observed functional difference between KIR2DL2/L3-E^35^ and -Q^35^ arises from the protein stability change caused by E35Q substitution.

## Discussion

KIR genes are highly polymorphic in nature exhibiting haplotypic and allelic variations^8^,^18^. The allelic diversity in each KIR gene ranges from 7 in KIR2DS3 to 76 in KIR3DL1^18^. Twelve KIR2DL2 alleles and 24 KIR2DL3 alleles have been identified thus far. Allelic variation may lead to functional diversity. For example a study by Moesta *et al.* reported that positions 16 and 148 accounted for KIR2DL2*001 being a stronger receptor for HLA-C ligand than KIR2DL3*001^9^. Schönberg K *et al.* showed that KIR2DL2 alleles might cross-react with HLA-C2 ligands but KIR2DL3 alleles could not^10^. A recent study by Fraizer *et al.* investigated the binding of 6 common KIR2DL2/L3 alleles with their HLA-C ligands^14^. They found that KIR2DL3*005 (which has proline and arginine at position 16 and 148 similar to those of KIR2DL3*001 but has glutamic acid at position 35 rather than glutamine) has affinity and avidity to HLA-C ligand as strong as KIR2DL2 alleles^14^. In line with these ligand-binding data, our functional cytotoxicity studies both *in vitro* and *in vivo* showed that KIR2DL2/L3 alleles with E^35^ are functionally stronger than Q^35^ alleles. NK cells licensed through stronger KIR2DL2/L3 alleles have higher cytotoxicity than those with weaker alleles against tumor cells with missing ligands, a finding in line with our previous observation that KIR2DL1^+^ NK cells licensed through stronger KIR2DL1-R^245^ have higher cytotoxicity than KIR2DL1-C^245 ^19,20.

KIR dimers are known to have higher affinity for HLA-C ligands than monomer. In the orthorhombic crystals of KIR2DL2, Snyder *et al.* found that two receptor molecules with the same orientation dimerize in such a way that the amino-terminal D1 domain of one receptor packs against the carboxyl-terminal D2 domain of the other molecule with their strands in an approximate orthogonal orientation, creating a D1/D2 heterodimer^21^. In another study, Fan *et al.* showed that covalently-linked KIR2DL1 dimer has higher affinity or avidity for HLA-Cw4 than monomer^22^. In the present study, molecular modeling analysis suggests that KIR2DL2/L3 alleles with the negatively charged E^35^ form electrostatic interactions with the positively charged H^55^ in KIR2DL2/L3 and thereby stabilizes the binding with HLA-C ligands. On the contrary, KIR2DL2/L3 encoded by Q^35^ alleles lack the electrostatic interactions and show much higher structural flexibility in the KIR2DL2/L3 dimer thus providing an explanation for the much weaker ligand binding and inhibitory function.

Previous studies have shown that differences in KIR gene content are associated with the risk of many human diseases, including autoimmune diseases, inflammatory disorders, infectious diseases, immunodeficiency, cancer, and reproductive disorders^23^. KIR2DL2/L3 is specifically known to be associated with Crohn’s disease^24^, multiple sclerosis^25^, chronic myeloid leukemia^26^, primary Sjogren’s syndrome^27^, lupus^28^, birdshot chorioretinopathy^29^, rheumatoid arthritis^30^, cervical intraepithelial neoplasia^31^, scleroderma^32^, and psoriasis^33^. KIR2DL2/L3 is also reported to be associated with herpes simplex virus 1^34^ as well as human immunodeficiency virus 1^35^ infections. Moreover, KIR2DL2/L3 are important determinant for donor selection in hematopoietic stem cell transplantation (HSCT). In this regard, we have previously showed that a single amino acid can be used to divide the KIR2DL1 alleles into functionally strong and weak groups^11^. In the allogeneic HSCT setting, this biologic difference can be translated to markedly different patient survival and risk of leukemia relapse^20^. Taken together, our findings herein will have significant implications in donor selection and outcome evaluation in HSCT and NK cell therapy (KIR2DL1-R^245^ and KIR2DL2/L3-E^35^ are predicted to be favorable). The results will also be useful in setting the framework for future NK cell research in various clinical settings, including susceptibility and outcome of cancers, pregnancy complications, chronic viral infections, and autoimmune diseases.

## Materials and Methods

### Statement

Animal experiment was carried out according to the protocols that were approved by The Institutional Animal Care and Use Committee of St. Jude Children’s Research Hospital.

### DNA constructs

Peripheral blood mononuclear cells were obtained from healthy human donors with informed consent under a protocol approved by the institutional review board at St Jude Children’s Research Hospital, in accordance with the Declaration of Helsinki. Total RNA was extracted from cells expressing KIR2DL2/L3 using RNA extraction kits (QIAGEN). cDNA of various alleles of KIR2DL2/L3 was generated from the RNA and cloned into mammalian expression vector pcDNA3 (Invitrogen). The identities of the KIR2DL2/L3 alleles were confirmed by sequencing. Specific amino acids were substituted into KIR2DL2/L3 using recombinant polymerase chain reaction. HLA-Cw7 cDNA was amplified from a normal human peripheral blood cDNA pool as described for KIR2DL2/L3 alleles, confirmed by sequencing, and cloned into retroviral vector MSCV-IRES-GFP (received from vector laboratory, St Jude Children’s Research Hospital).

### Cell lines, culture, and transfection

YT-Indy cells (a generous gift from Dr. Zacharie Brahmi, Indiana University) were cultured in RPMI 1640 supplemented with 10% fetal bovine serum (FBS), 1 mM penicillin/streptomycin, 2 mM l-glutamine, 1 mM sodium pyruvate, and 1% minimum essential medium nonessential amino acids (Invitrogen). B-lymphoblastic cell line 721.221 was purchased from the International Histocompatibility Working Group and cultured in RPMI 1640 supplemented with 20% FBS and 1 mM penicillin/streptomycin. YT-Indy cells were transfected with pcDNA3 vector containing various KIR2DL2/L3 alleles by electroporation (Gene Pulser II; Bio-Rad). Stable cell lines were generated by selection in Geneticin (Invitrogen). The 721.221 cells were transduced with retroviral vector MSCV-IRES-GFP containing HLA-Cw7. High-expressing cells was sorted by flow cytometric cell sorting using the monoclonal antibody (mAb) against HLA-C (One Lambda).

### KIR2DL2/L3 single nucleotide polymorphism (SNP) and genotyping assay

To detect the presence of various alleles of *KIR2DL2/L3* with glutamic acid or glutamine at position 35, a single-nucleotide mismatch detection assay was developed as described previously^19^,^36^. Briefly, primers for the assay were designed in such a way that they amplified all the alleles of the *KIR2DL2/L3* gene as well as the amplicon containing the polymorphic region of interest. The forward primer was 5′-CATCCTGCAATGTTGGTCAG-3′ and the reverse primer was 5′-CAAGGTCTTGCATCATGGGA-3′. The probe for *KIR2DL2/L3* alleles with a glutamic acid at position 35 was 6Fam-CAGGTTTGAGCACT-MGBNFQ and for those with a glutamine at the same position was VIC-CAGGTTTCAGCACT-MGBNFQ. Each assay reaction mix contained a 250 nM probe concentration and 100 ng of genomic DNA in 1× TaqMan genotyping master mix (Applied Biosystems). The assay was performed on an HT7900 Sequence Detection System (Applied Biosystems) following the allelic discrimination assay protocol provided by the manufacturer. KIR2DL2/L3 genotyping was performed by a PCR-SSP method as described previously^17^.

### Flow cytometry

The expression of KIR2DL2/L3 and other NK cell receptors were analyzed using flow cytometry. The following clones of antibody were used for phenotypic analysis: anti-CD158ah (11PB6, EB6.B), anti-CD158b (CH-L, GL183), anti-CD158e (DX9), anti-DNAM-1 (DX11), anti-CD11a (HI111), anti-NTBA (292811), anti-CD244, anti-CD3 (SK7, UCHT1), anti-granzyme B (GB10), anti-NKG2a (Z199), anti-NKp30 (Z25), anti-NKp44 (Z231), anti-NKp46 (BAB281), anti-NKG2D (1D11), anti-CD56 (MY31, N901), and anti-CD14 (MphiP9). Flow cytometry analyses were conducted with LSRII (BD Biosciences) and FlowJo 8.8.6 software (Tree Star).

### Cytotoxicity assay

To determine the functional differences amongst different KIR2DL2/L3 and mutated alleles, cytotoxic activity was measured by using the DELFIA BATDA reagent (PerkinElmer Life and Analytical Sciences) following the manufacturer’s instruction. BATDA-labeled B-lymphoblastic cell lines 721.221 with or without HLA-C ligands were used as target cells at an effector : target (E:T) ratio of 20:1 for 2 hours at 37 °C. The fluorescence signals were measured using a Wallac Victor 2 Counter Plate Reader (PerkinElmer Life and Analytical Sciences).

### *CD107* degranulation assay

NK cells were tested for their cytolytic potential with the CD107 degranulation assay. Effector cells were cocultured with target cells at 1:1 ratio in the presence of anti-CD107-FITC or -APC antibodies. After 1 hour of co-culture, GolgiStop (BD Biosciences) was added and the cells were incubated for 4 more hours. The cells were then harvested, stained, and analyzed for CD107.

### *In vivo* experiments

Non-obese diabetic/severe combined immunodeficient (NOD/SCID) IL-2^γc−/−^ mice, 8- to 12-weeks old, were used as animal model. 721.221-Cw7-luciferase cells were used as target cells. Mice were γ-irradiated at a dose of 200 cGy 1 day prior to intraperitoneall injections of 5 × 10^5^ 721.221-Cw7 cells. On the following day, 5 × 10^6^ YT-Indy cells expressing different KIR2DL2/L3 alleles (effector cells) were injected intraperitoneally (E:T = 10:1). Disease progression in the injected mice was monitored by bioluminescence imaging (Xenogen, PerkinElmer Life and Analytical Sciences). The mice were sacrificed when they displayed signs of significant tumor progression. The experiments were terminated after 3 weeks as bioluminescence reached saturation point.

### Molecular modeling

The structural effects of E35Q mutation of KIR2DL2 were simulated by molecular dynamics simulation^37^. The dimer structure of both E35 and Q35 were derived from crystal structure of KIR2DL2-HLA complex (PDB ID: 1EFX)^21^,^38^. The TIP3P water solvent box with 10 angstroms from the protein were added to each system, and counterions were used for neutralizing the charges. Each system were thoroughly minimized and heated to 300 K, and simulated for 25 ns by Langevin dynamics. The backbones root mean squire fluctuations (RMSFs) of CA atoms were calculated to evaluate protein stabilities.

### Statistical analysis

Statistical significance between 2 groups was calculated using Student T test. The nominal significance level was set at 0.05.

## Additional Information

**How to cite this article**: Bari, R. *et al.* KIR2DL2/2DL3-E^35^ alleles are functionally stronger than -Q^35^ alleles. *Sci. Rep.* **6**, 23689; doi: 10.1038/srep23689 (2016).

## Supplementary Material

Supplementary Information

## Figures and Tables

**Table 1:** KIR2DL2/L3 alleles are grouped based on the presence of glutamic acid or glutamine at position 35.

**	Alleles	D1				D2								ST		TM						CYT										
	E^35^	11	16	35	41	50	114	123	131	148	167	200	208	216	221	225	232	236	239	245	248	254	260	266	268	269	272	282	296	298	312	320
32.0	L2*001	L	R	E	R	H	P	S	R	C	G	I	P	K	R	I	V	F	de	R	S	A	S	A	S	E	D	T	R	S	A	N
0	L2*002	–	–	–	–	–	–	–	–	–	–	–	–	–	–	–	–	–	–	–	–	–	–	–	R	Q	-	–	–	–	–	–
20.2	L2*003	–	–	–	–	–	–	–	–	–	–	T	–	–	–	–	–	–	–	–	–	–	–	–	–	–	–	–	–	–	T	–
0	L2*005	–	–	–	–	–	–	–	–	–	–	–	–	–	–	–	–	–	–	–	–	–	–	–	–	–	–	–	–	–	T	–
0	L2*006	–	P	–	–	–	–	–	–	–	–	T	–	–	–	–	–	–	–	–	–	–	–	–	–	–	–	–	–	–	T	–
NA	L2*007	–	–	–	–	–	–	–	–	–	–	–	–	–	–	–	–	A	–	–	–	–	–	–	–	–	–	–	–	–	–	–
NA	L2*008	–	–	–	–	–	–	–	–	–	–	–	–	–	–	–	–	–	–	–	C	–	–	–	–	–	–	–	–	–	T	–
NA	L2*009	–	–	–	–	–	–	–	–	R	–	T	–	–	–	–	–	–	–	–	–	–	–	–	–	–	–	–	–	–	T	–
NA	L2*010	–	–	–	–	–	–	–	–	–	–	–	–	–	–	–	–	–	–	–	C	–	–	–	–	–	Y	–	–	–	T	–
0	L3*004	R	P	–	–	R	–	–	–	R	–	T	–	–	I	V	–	–	L	–	C	V	P	V	R	–	–	A	H	–	T	
7.8	L3*005	R	P	–	–	R	–	–	–	R	–	T	–	E	–	V	–	–	L	–	C	V	P	V	R	–	–	A	H	–	T	
NA	L3*010	R	P	–	–	R	–	–	–	R	–	T	–	E	–	V	–	V	L	–	C	V	P	V	R	–	–	A	–	–	T	#
NA	L3*014	–	P	–	–	R	–	–	–	R	–	T	–	E	–	V	–	–	L	–	C	V	P	V	R	–	–	A	–	–	T	
NA	L3*015	–	P	–	–	–	–	–	–	R	–	T	L	E	–	V	–	–	L	–	C	V	P	V	R	–	–	A	H	–	T	
NA	L3*016	–	P	–	–	–	–	N	–	R	–	T	–	E	–	V	–	–	L	–	C	V	P	V	R	–	–	A	–	–	T	
NA	L3*017	–	P	–	–	R	–	–	–	R	–	T	–	E	–	V	–	–	L	–	C	V	P	V	R	–	–	A	–	–	T	#
	Q^35^																															
1.7	L2*004	–	P	Q	T	–	–	–	–	–	D	T	–	E	–	–	–	–	–	–	–	–	–	–	I	–	–	–	H	–	T	S
71.6	L3*001	–	P	Q	–	–	–	–	–	R	–	T	–	E	–	V	–	–	L	–	C	V	P	V	R	–	–	A	–	–	T	
35.6	L3*002	–	P	Q	–	–	–	–	–	R	–	T	L	E	–	V	–	–	L	–	C	V	P	V	R	–	–	A	H	–	T	
1.1	L3*003	–	P	Q	–	–	–	–	–	R	–	T	–	E	–	V	–	–	L	–	C	V	P	V	R	–	–	T	–	–	T	
1.1	L3*006	–	P	Q	–	–	–	–	–	R	–	T	–	E	–	V	–	–	L	–	C	V	P	V	R	–	–	A	H	–	T	
NA	L3*007	–	P	Q	–	–	–	–	–	R	–	T	L	E	–	V	–	–	L	–	C	–	–	–	–	–	–	A	H	–	T	
NA	L3*009	–	P	Q	–	–	–	–	–	R	–	T	–	E	–	V	–	–	L	–	C	V	P	V	R	–	–	A	–	–	T	
NA	L3*011	–	P	Q	–	L	–	–	–	R	–	T	–	E	–	V	–	–	L	–	C	V	P	V	R	–	–	A	–	–	T	
NA	L3*012	–	P	Q	–	–	–	–	–	R	–	T	–	E	–	V	–	–	L	–	C	–	P	V	R	–	–	A	H	F	T	
NA	L3*013	–	P	Q	–	–	–	–	Q	R	–	T	–	E	–	V	–	–	L	–	C	P	P	V	R	–	–	A	–	–	T	

**indicates percentage of individuals that have the KIR allele in Brazil Belo Horizonte Caucasian (http://www.allelefrequencies.net/kir6002a.asp). “–” indicates identity with consensus to KIR2DL2*001; #alleles has few more amino acids “RSKVV SCPX”.
