# Supplementary material for: KIR2DL2/2DL3-E35 alleles are functionally stronger than -Q35 alleles
Source: Sci Rep. 2016 Mar 31;6:23689. doi: 10.1038/srep23689 (PMC4814820; doi:10.1038/srep23689)
Supplement: Supplementary Information [file srep23689-s1.doc]

**KIR2DL2/2DL3-E^35^ alleles are functionally stronger than -Q^35^ alleles.**

Rafijul Bari^1^, Rajoo Thapa^1^, Ju Bao^2^, Ying Li^1^, Jie Zheng^2^, Wing Leung^1,3*^.


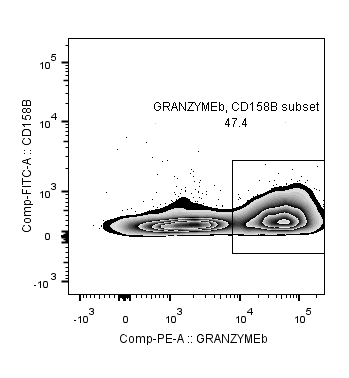

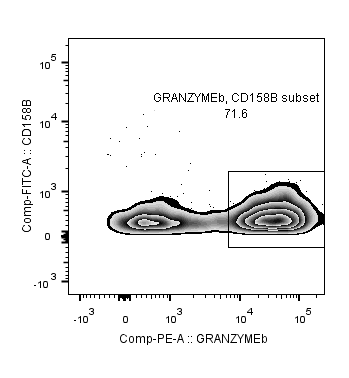

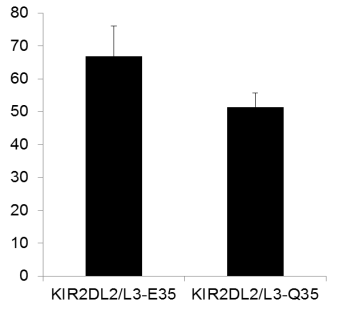

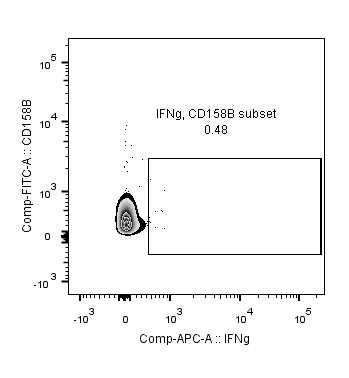

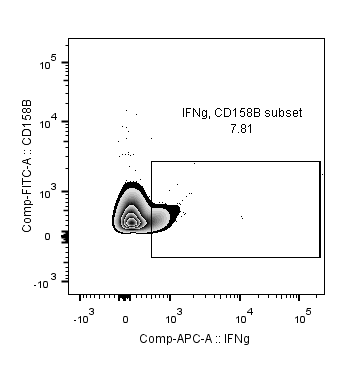

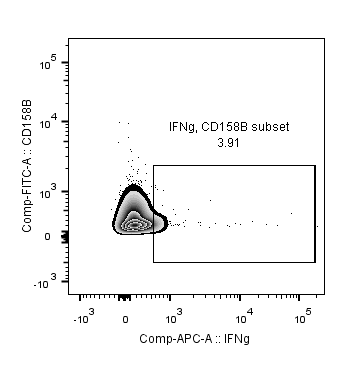

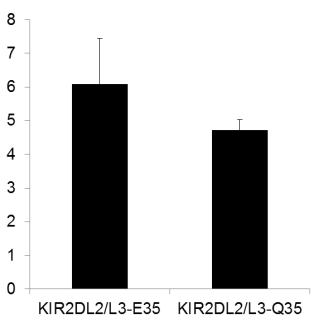


**Granzyme B**

***P = 0.01***

***P = 0.04***

**IFN-γ**

**A**

**B**

**Supplementary Figure S1.** Donor PBMCs were stimulated with MHC class I deficient cell line 721.221. KIR2DL2/L3^+^ NK cell subsets were gated, and (A) granzyme B and (B) IFNγ productions were detected using specific antibodies. Figures are representative of four individual donors. Bar chart at the right hand average summary. Error bars represent SD and p values were determined by student t-test.

**Isotype**

**KIR2DL2/L3-E^35^**

**KIR2DL2/L3-Q^35^**

**Isotype**

**KIR2DL2/L3-E^35^**

**KIR2DL2/L3-Q^35^**


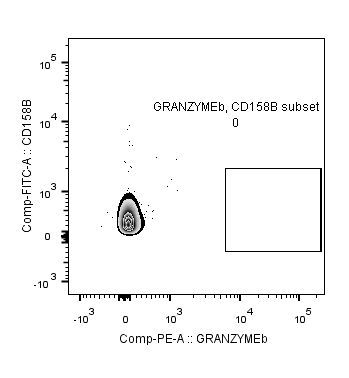


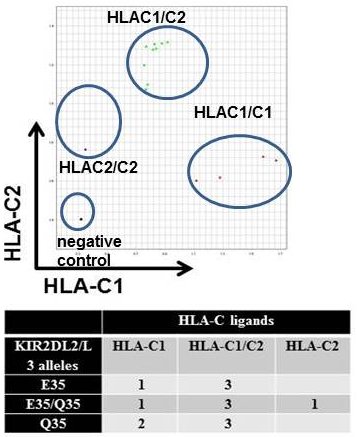


**Supplementary Figure S2.** Donors HLA-C ligands were typed using single nucleotide polymorphism assay. Upper part of the figure is allelic discrimination plot; the lower panel is shows the summary results


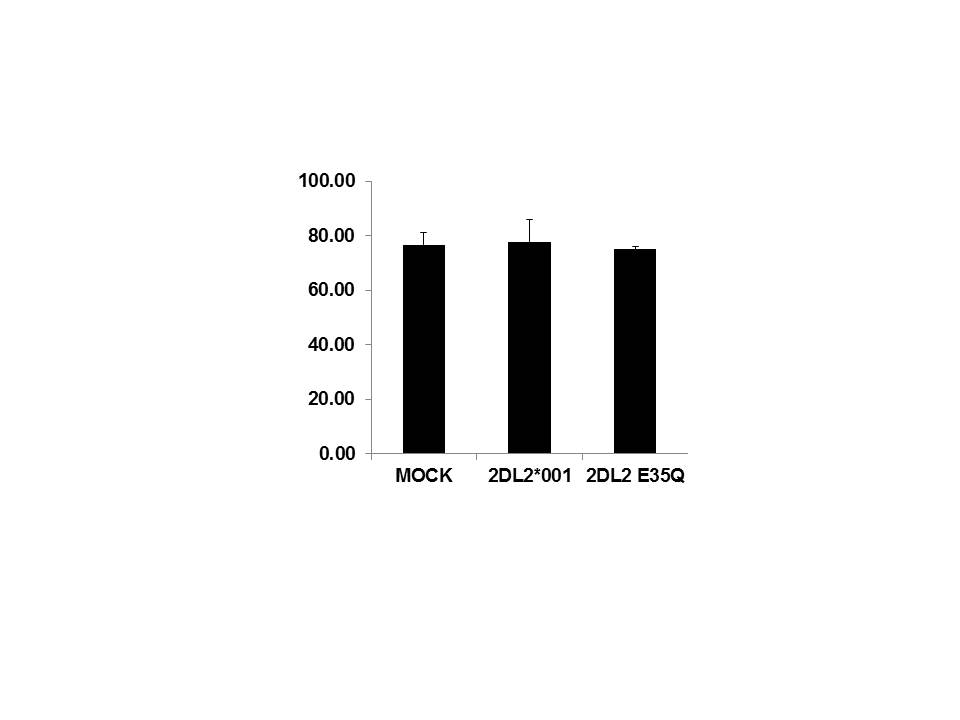


**Supplementary Figure S3.** NK cell line YT-Indy was transduced with vector (mock), KIR2DL2*001 (2DL2*001), and KIR2DL2*001 mutant (2DL2 E35Q). Specific killing was assessed against 721.221 by BADTA release assay. The experiments were repeated for 3 times. Error bars represent SD.
